# Robust generation of entangled state via ground-state antiblockade of Rydberg atoms

**DOI:** 10.1038/s41598-017-16533-9

**Published:** 2017-11-28

**Authors:** Y. J. Zhao, B. Liu, Y. Q. Ji, S. Q. Tang, X. Q. Shao

**Affiliations:** 1 Center for Quantum Sciences and School of Physics, Northeast Normal University, Changchun, 130024 People’s Republic of China; 2 Center for Advanced Optoelectronic Functional Materials Research, and Key Laboratory for UV Light-Emitting Materials and Technology of Ministry of Education, Northeast Normal University, Changchun, 130024 People’s Republic of China; 3 Department of Physics and Electronic Information Science, Hengyang Normal University, Hengyang, 421008 People’s Republic of China

## Abstract

We propose a mechanism of ground-state antiblockade of Rydberg atoms, which is then exploited to prepare two-atom entangled state via three different kinds of pulses. First we use the pulses in the form of sin^2^ and cos^2^ functions and obtain a maximally entangled state at an accurate interaction time. Then the method of stimulated Raman adiabatic passage (STIRAP) is adopted for the entanglement generation, which is immune to the fluctuations of revelent parameters but requires a long time. Finally we capitalize the advantages of the former two methods and employ shortcuts to adiabatic passage (STAP) to generate the maximal entanglement. The strictly numerical simulation reveals that the current scheme is robust against spontaneous emission of atoms due to the virtual excitation of Rydberg states, and all of the above methods favor a high fidelity with the present experimental technology.

## Introduction

Quantum entanglement, referring to the non-local and non-classical strong correlations between individual quantum objects, such as atoms, ions, superconducting circuits, spins, or photons, is one of the most distinct features in quantum mechanics and an important resource in quantum information and quantum metrology. It has been widely used in quantum teleportation^1–3^, quantum cryptography^4,5^, quantum dense coding^6,7^, quantum secure direct communication^8–10^ and quantum key distribution^11–13^ etc. Owing to its importance, the entangled states have become a hot research topic in recent decades.

As an attractive system for manipulation of quantum information, neutral atoms are similar to ions, the best developed system to date, due to their long-lived hyperfine states that are robust against decoherence, and they can be precisely manipulated by optical and other electromagnetic fields. In addition, when the neutral atoms are excited to the Rydberg states, it will exhibit large dipole moments resulting in a dipole-dipole interaction which is strong enough to shift the atomic energy levels and prevent more than one atom from being excited to the Rydberg state^14–19^, which is related to Rydberg blockade phenomenon. Recently, the blockade between two atoms set about 4 *μ*m and 10 *μ*m apart were reported independently by two experimental groups^20,21^. Subsequently, many proposals were presented to prepare entanglement with Rydberg blockade^22–28^. For example, Saffman *et al*. produced *N*-particle entangled states using Rydberg blockade interactions and predicted that eight-atom entangled states can be produced with a fidelity of 84% in cold Rb atoms^23^. Wilk *et al*. reported the generation of entanglement between two individual ^87^Rb atoms in hyperfine ground states which are held in two optical tweezers separated by 4 *μ*m relying on the Rydberg blockade effect^24^. Maller *et al*. performed experiments in an array of single Cs atom qubits with a site to site spacing of 3.8 *μ*m and created Bell states using the standard protocol with a Rydberg-blockade controlled-z gate and single qubit operations^27^.

In certain configurations, the blockade effect can be overcome and atom pairs can selectively be excited at short distance. This so-called antiblockade was initially proposed by Ates *et al*.^29^ for a three-level two-photon Rydberg excitation scheme and it has been studied and applied for preparation of entanglement theoretically^30,31^. In short, by adjusting the distance between Rydberg atoms in a controllable way, the blockade effect and the antiblockade effect can be preferred or suppressed, which is of particular interest for quantum information.

However, it should be noted that the populations of the excited Rydberg states will decrease the fidelity of entangled state due to the spontaneous emission of atoms since the lifetime of Rydberg state is finite. Very recently, Shao *et al*. put forward an efficient scheme of ground-state blockade for *N*-type Rydberg atoms by virtue of Rydberg antiblockade effect and Raman transition^32^, which averts the spontaneous emission of the excited Rydberg state, and keep the nonlinear Rydberg-Rydberg interaction (RRI) at the same time. Inspired by this scheme, in this paper, we propose a mechanism of ground-state antiblockade for Rydberg atoms, i.e., the effectively coherent Rabi oscillation between two ground states $|gg\rangle$ and $|ee\rangle$ can be achieved. As its application, we will explore three ways to implement the two-atom maximally entangled state. First, we adopt the pulses in the form of sin^2^ and cos^2^ functions and obtain a high-fidelity maximally entangled state at an accurate interaction time. The second method takes advantage of STIRAP which is insensitive to parameter fluctuations but needs a relatively long time. Finally, we use the shortcuts to adiabatic passage which combines the former two methods’ advantages to generate entangled state. The prominent advantage of our scheme is that the quantum information is encoded into the ground states of Rydberg atoms, and the evolution process of system is robust against atomic decay for two-atom entangled state preparation.

## Theoretical Model

**Figure 1:**
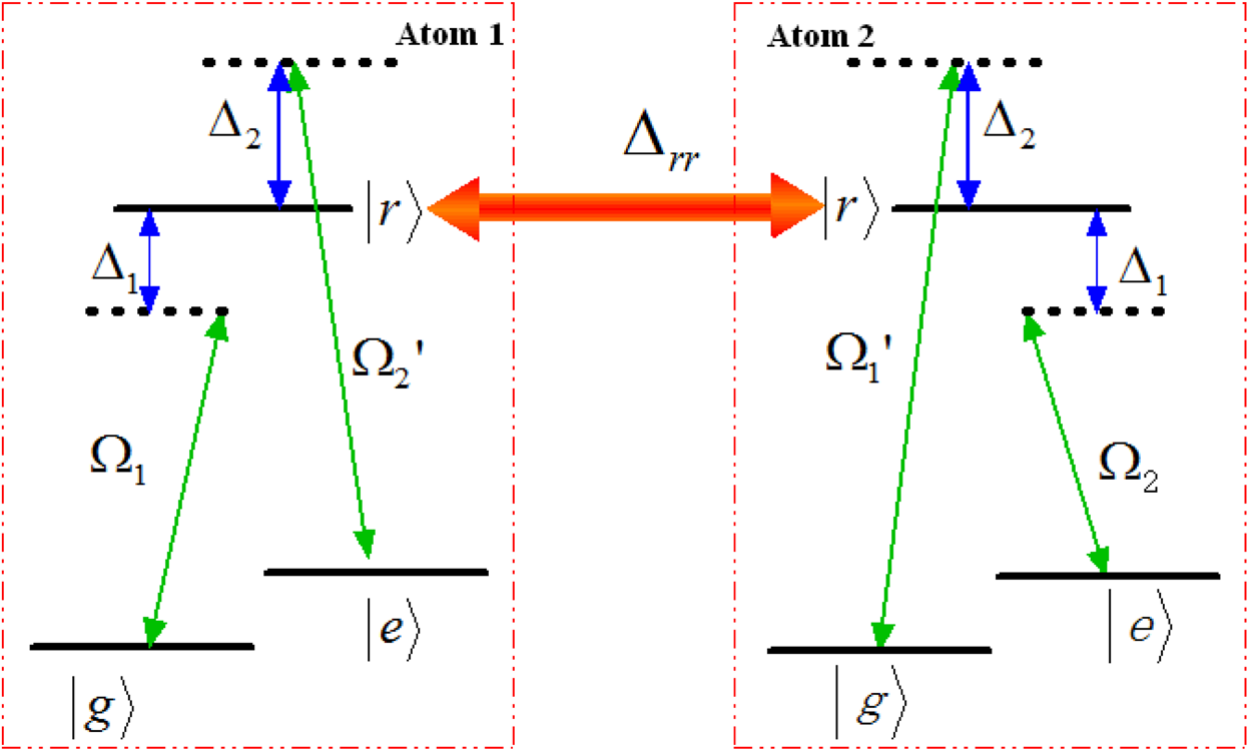
Schematic view of atomic-level configuration. $|g\rangle$ and $|e\rangle$ are the hyperfine states in the ground-state manifold, respectively, and $|r\rangle$ state is excited Rydberg state. ${{\rm{\Delta }}}_{rr}$ denotes the RRI strength. Here we adopted four pulses Ω_1_, Ω_1_, ${{\rm{\Omega }}}_{1}^{^{\prime} }$, ${{\rm{\Omega }}}_{2}^{^{\prime} }$.

As shown in Fig. 1, we consider two identical Rydberg atoms trapped in two separate microscopic dipole traps. The states $|g\rangle$ and $|e\rangle$ are the hyperfine states in the ground-state manifold, respectively, and state $|r\rangle$ is the excited Rydberg state. One atomic transition $|g\rangle (|e\rangle )\leftrightarrow |r\rangle$ is driven by a classical laser field with Rabi frequency ${{\rm{\Omega }}}_{1}({{\rm{\Omega }}}_{2}^{^{\prime} })$, detuned by ${{\rm{\Delta }}}_{1}$(−${{\rm{\Delta }}}_{2}$), the other atomic transition $|g\rangle (|e\rangle )\leftrightarrow |r\rangle$ is driven by a classical laser field with Rabi frequency ${{\rm{\Omega }}}_{1}^{^{\prime} }({{\rm{\Omega }}}_{2})$ and the corresponding detuning is $-{{\rm{\Delta }}}_{2}$(${{\rm{\Delta }}}_{1}$). The Hamiltonian of the whole system can be written as

1 $$\begin{array}{c}{\hat{H}}_{I}={{\rm{\Omega }}}_{1}|r{\rangle }_{1}{\langle g|{e}^{i{{\rm{\Delta }}}_{1}t}+{{\rm{\Omega }}}_{2}^{^{\prime} }|r\rangle }_{1}\langle e|{e}^{-i{{\rm{\Delta }}}_{2}t}\\ \quad \quad \,+{{\rm{\Omega }}}_{1}^{^{\prime} }|r{\rangle }_{2}{\langle g|{e}^{-i{{\rm{\Delta }}}_{2}t}+{{\rm{\Omega }}}_{2}|r\rangle }_{2}\langle e|{e}^{i{{\rm{\Delta }}}_{1}t}+{\rm{H}}\mathrm{.}{\rm{c}}\mathrm{.}+U|rr\rangle \langle rr|,\end{array}$$

where *U* is the RRI strength which relates to the principal quantum numbers and the distance between the Rydberg atoms. To see clearly the roles of the RRI term, we rewrite the full Hamiltonian with the two-atom basis {$|gg\rangle$, $|ge\rangle$, $|gr\rangle$, $|eg\rangle$, $|ee\rangle$, $|er\rangle$, $|rg\rangle$, $|re\rangle$, $|rr\rangle$} and move to a rotation frame with respect to $\exp (-iU|rr\rangle \langle rr|t)$. Then we have

2 $$\begin{array}{rcl}{\hat{H}}_{IR} & = & {{\rm{\Omega }}}_{1}{e}^{i{{\rm{\Delta }}}_{1}t}(|rg\rangle \langle gg|+|re\rangle \langle ge|+{e}^{iUt}|rr\rangle \langle gr|)\\  &  & +{{\rm{\Omega }}}_{2}^{^{\prime} }{e}^{-i{{\rm{\Delta }}}_{2}t}(|rg\rangle \langle eg|+|re\rangle \langle ee|+{e}^{iUt}|rr\rangle \langle er|)\\  &  & +{{\rm{\Omega }}}_{1}^{^{\prime} }{e}^{-i{{\rm{\Delta }}}_{2}t}(|gr\rangle \langle gg|+|er\rangle \langle eg|+{e}^{iUt}|rr\rangle \langle rg|)\\  &  & +{{\rm{\Omega }}}_{2}{e}^{i{{\rm{\Delta }}}_{1}t}(|gr\rangle \langle ge|+|er\rangle \langle ee|+{e}^{iUt}|rr\rangle \langle re|)+{\rm{H}}\mathrm{.}{\rm{c}}\mathrm{..}\end{array}$$

Now we adjust the classical field and RRI strength to satisfy $U=\delta +({{\rm{\Delta }}}_{2}-{{\rm{\Delta }}}_{1})$. On account of the large detuning condition ${{\rm{\Delta }}}_{1}({{\rm{\Delta }}}_{2})\gg {{\rm{\Omega }}}_{1}({{\rm{\Omega }}}_{2},\,{{\rm{\Omega }}}_{1}^{^{\prime} },\,{{\rm{\Omega }}}_{2}^{^{\prime} })$, we may safely eliminate the high-frequency oscillating terms and obtain

3 $$\begin{array}{rcl}{\hat{H}}_{E} & = & (\frac{{{\rm{\Omega }}}_{1}^{^{\prime} 2}}{{{\rm{\Delta }}}_{2}}-\frac{{{\rm{\Omega }}}_{1}^{2}}{{{\rm{\Delta }}}_{1}})|gg\rangle \langle gg|+(\frac{{{\rm{\Omega }}}_{2}^{^{\prime} 2}}{{{\rm{\Delta }}}_{2}}-\frac{{{\rm{\Omega }}}_{2}^{2}}{{{\rm{\Delta }}}_{1}})|ee\rangle \langle ee|\\  &  & +(\frac{{{\rm{\Omega }}}_{1}^{2}}{{{\rm{\Delta }}}_{1}}-\frac{{{\rm{\Omega }}}_{1}^{^{\prime} 2}}{{{\rm{\Delta }}}_{2}}+\frac{{{\rm{\Omega }}}_{2}^{2}}{{{\rm{\Delta }}}_{1}}-\frac{{{\rm{\Omega }}}_{2}^{^{\prime} 2}}{{{\rm{\Delta }}}_{2}})|rr\rangle \langle rr|\\  &  & +[(\frac{{{\rm{\Omega }}}_{2}{{\rm{\Omega }}}_{2}^{^{\prime} }}{{{\rm{\Delta }}}_{2}}-\frac{{{\rm{\Omega }}}_{2}{{\rm{\Omega }}}_{2}^{^{\prime} }}{{{\rm{\Delta }}}_{1}}){e}^{i\delta t}|rr\rangle \langle ee|\\  &  & +(\frac{{{\rm{\Omega }}}_{1}{{\rm{\Omega }}}_{1}^{^{\prime} }}{{{\rm{\Delta }}}_{2}}-\frac{{{\rm{\Omega }}}_{1}{{\rm{\Omega }}}_{1}^{^{\prime} }}{{{\rm{\Delta }}}_{1}}){e}^{i\delta t}|rr\rangle \langle gg|+{\rm{H}}\mathrm{.}{\rm{c}}\mathrm{.}],\end{array}$$

where $({{\rm{\Omega }}}_{1}^{^{\prime} 2}{/{\rm{\Delta }}}_{2}-{{\rm{\Omega }}}_{1}^{2}{/{\rm{\Delta }}}_{1})$, $({{\rm{\Omega }}}_{2}^{^{\prime} 2}{{\rm{\Delta }}}_{2}-{{\rm{\Omega }}}_{2}^{2}{/{\rm{\Delta }}}_{1})$, $({{\rm{\Omega }}}_{1}^{2}{/{\rm{\Delta }}}_{1}-{{\rm{\Omega }}}_{1}^{^{\prime} 2}{/{\rm{\Delta }}}_{2}+{{\rm{\Omega }}}_{2}^{2}{/{\rm{\Delta }}}_{1}-{{\rm{\Omega }}}_{2}^{^{\prime} 2}{/{\rm{\Delta }}}_{2})$ originate from the Stark shifts of states $|gg\rangle$, $|ee\rangle$, $|rr\rangle$, respectively. And $({{\rm{\Omega }}}_{2}{{\rm{\Omega }}}_{2}^{^{\prime} }{/{\rm{\Delta }}}_{2}-{{\rm{\Omega }}}_{2}{{\rm{\Omega }}}_{2}^{^{\prime} }{/{\rm{\Delta }}}_{1}){e}^{-i\delta t}$, $({{\rm{\Omega }}}_{1}{{\rm{\Omega }}}_{1}^{^{\prime} }{/{\rm{\Delta }}}_{2}-{{\rm{\Omega }}}_{1}{{\rm{\Omega }}}_{1}^{^{\prime} }{/{\rm{\Delta }}}_{1}){e}^{-i\delta t}$ are the effective coupling strength between $|rr\rangle$ and $|ee\rangle (|gg\rangle )$. We can further eliminate Stark-shift terms of ground states with the help of auxiliary levels. Hence Eq. (3) can be rewritten as

4 $$\begin{array}{rcl}{\hat{H}}_{E}^{^{\prime} } & = & (\frac{{{\rm{\Omega }}}_{1}^{2}}{{{\rm{\Delta }}}_{1}}-\frac{{{\rm{\Omega }}}_{1}^{^{\prime} 2}}{{{\rm{\Delta }}}_{2}}+\frac{{{\rm{\Omega }}}_{2}^{2}}{{{\rm{\Delta }}}_{1}}-\frac{{{\rm{\Omega }}}_{2}^{^{\prime} 2}}{{{\rm{\Delta }}}_{2}})|rr\rangle \langle rr|\\  &  & +[(\frac{{{\rm{\Omega }}}_{2}{{\rm{\Omega }}}_{2}^{^{\prime} }}{{{\rm{\Delta }}}_{2}}-\frac{{{\rm{\Omega }}}_{2}{{\rm{\Omega }}}_{2}^{^{\prime} }}{{{\rm{\Delta }}}_{1}}){e}^{i\delta t}|rr\rangle \langle ee|\\  &  & +(\frac{{{\rm{\Omega }}}_{1}{{\rm{\Omega }}}_{1}^{^{\prime} }}{{{\rm{\Delta }}}_{2}}-\frac{{{\rm{\Omega }}}_{1}{{\rm{\Omega }}}_{1}^{^{\prime} }}{{{\rm{\Delta }}}_{1}}){e}^{i\delta t}|rr\rangle \langle gg|+{\rm{H}}\mathrm{.}{\rm{c}}\mathrm{.}]\mathrm{.}\end{array}$$

For simplicity, we have set ${{\rm{\Omega }}}_{1}^{2}{/{\rm{\Delta }}}_{1}-{{\rm{\Omega }}}_{1}^{^{\prime} 2}{/{\rm{\Delta }}}_{2}+{{\rm{\Omega }}}_{2}^{2}{/{\rm{\Delta }}}_{1}-{{\rm{\Omega }}}_{2}^{^{\prime} 2}{/{\rm{\Delta }}}_{2}=\delta ^{\prime}$, ${{\rm{\Omega }}}_{1}{{\rm{\Omega }}}_{1}^{^{\prime} }{/{\rm{\Delta }}}_{2}-{{\rm{\Omega }}}_{1}{{\rm{\Omega }}}_{1}^{^{\prime} }{/{\rm{\Delta }}}_{1}={{\rm{\Omega }}}_{a}$ and ${{\rm{\Omega }}}_{2}{{\rm{\Omega }}}_{2}^{^{\prime} }{/{\rm{\Delta }}}_{2}-{{\rm{\Omega }}}_{2}{{\rm{\Omega }}}_{2}^{^{\prime} }{/{\rm{\Delta }}}_{1}={{\rm{\Omega }}}_{b}$. After a unitary transformation $\hat{S}=\exp (-i\delta t|rr\rangle \langle rr|)$ removing the time-dependent terms, Eq. (4) becomes

5 $${\hat{H}}_{ER}^{^{\prime} }={{\rm{\Omega }}}_{a}|rr\rangle \langle gg|+{{\rm{\Omega }}}_{b}|rr\rangle \langle ee|+{\rm{H}}{\rm{.c}}{\rm{.}}+(\delta ^{\prime} +\delta )|rr\rangle \langle rr\mathrm{|.}$$

**Figure 2:**
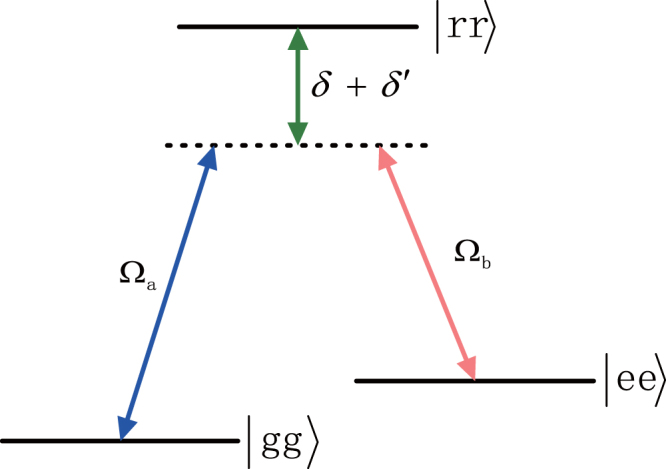
The atomic level configuration for the effective Hamiltonian, where the effective Rabi frequencies ${{\rm{\Omega }}}_{a}={{\rm{\Omega }}}_{1}{{\rm{\Omega }}}_{1}^{^{\prime} }{/{\rm{\Delta }}}_{2}-{{\rm{\Omega }}}_{1}{{\rm{\Omega }}}_{1}^{^{\prime} }{/{\rm{\Delta }}}_{1}$, ${{\rm{\Omega }}}_{b}={{\rm{\Omega }}}_{2}{{\rm{\Omega }}}_{2}^{^{\prime} }{/{\rm{\Delta }}}_{2}-{{\rm{\Omega }}}_{2}{{\rm{\Omega }}}_{2}^{^{\prime} }{/{\rm{\Delta }}}_{1}$, and $\delta ^{\prime} ={{\rm{\Omega }}}_{1}^{2}{/{\rm{\Delta }}}_{1}-{{\rm{\Omega }}}_{1}^{^{\prime} 2}{/{\rm{\Delta }}}_{2}+{{\rm{\Omega }}}_{2}^{2}{/{\rm{\Delta }}}_{1}-{{\rm{\Omega }}}_{2}^{^{\prime} 2}{/{\rm{\Delta }}}_{2}$.

We can deem Hamiltonian of Eq. (5) an effective Λ-type three-level system with an excited state $|rr\rangle$ and two ground states $|gg\rangle$ and $|ee\rangle$ as shown in Fig. 2. For this effective Hamiltonian, the transition $|gg\rangle (|ee\rangle )\leftrightarrow |rr\rangle$ is driven by a classical laser field with Rabi frequency ${{\rm{\Omega }}}_{a}({{\rm{\Omega }}}_{b})$. ($\delta ^{\prime} +\delta$) represents the corresponding detuning parameter. By adiabatically eliminating the state $|rr\rangle$ under the condition $\nu =\delta ^{\prime} +\delta \gg \{{{\rm{\Omega }}}_{a},{{\rm{\Omega }}}_{b}\}$, we have the final effective Hamiltonian

6 $${\hat{H}}_{F}=\frac{{{\rm{\Omega }}}_{a}{{\rm{\Omega }}}_{b}}{\nu }|gg\rangle \langle ee|+{\rm{H}}{\rm{.c}}\mathrm{.,}$$

where the Stark-shift terms originating from the two-photon transition are disregarded. It should be noted that in order to obtain this kind of spin squeezing-like Hamiltonian, six lasers were applied by Bouchoule *et al*.^33^, however, four lasers are enough in our proposal.

**Figure 3:**
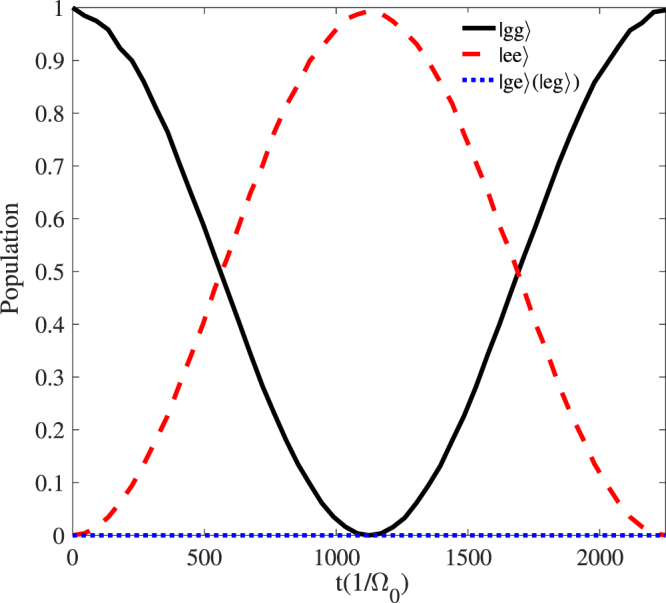
Time evolution of the populations for the states $|gg\rangle$, $|ee\rangle$, $|ge\rangle$ and $|eg\rangle$ by setting ${{\rm{\Omega }}}_{1}^{^{\prime} }{/{\rm{\Omega }}}_{0}={{\rm{\Omega }}}_{2}^{^{\prime} }{/{\rm{\Omega }}}_{0}=$ ${{\rm{\Omega }}}_{1}{/{\rm{\Omega }}}_{0}={{\rm{\Omega }}}_{2}{/{\rm{\Omega }}}_{0}=1$, ${{\rm{\Delta }}}_{1}{/{\rm{\Omega }}}_{0}=20$, ${{\rm{\Delta }}}_{2}{/{\rm{\Omega }}}_{0}=70$, and $\delta {/{\rm{\Omega }}}_{0}=1$ governed by the original Hamiltonian ${\hat{H}}_{I}$.

In Fig. 3, we plot the populations of states $|gg\rangle$, $|ee\rangle$, $|ge\rangle$ and $|eg\rangle$ by setting ${{\rm{\Omega }}}_{1}^{^{\prime} }{/{\rm{\Omega }}}_{0}={{\rm{\Omega }}}_{2}^{^{\prime} }{/{\rm{\Omega }}}_{0}=$ ${{\rm{\Omega }}}_{1}{/{\rm{\Omega }}}_{0}={{\rm{\Omega }}}_{2}{/{\rm{\Omega }}}_{0}=1$, ${{\rm{\Delta }}}_{1}{/{\rm{\Omega }}}_{0}=20$, ${{\rm{\Delta }}}_{2}{/{\rm{\Omega }}}_{0}=70$, $\delta {/{\rm{\Omega }}}_{0}=1$, $U=({{\rm{\Delta }}}_{2}-{{\rm{\Delta }}}_{1}+\delta )$ governed by the original Hamiltonian ${\hat{H}}_{I}$. It shows that the ground state $|gg\rangle$ resonantly interacts with the ground state $|ee\rangle$ under the condition of large detuning and there is nearly no population for the states $|ge\rangle$ or $|eg\rangle$. In addition, from the Hamiltonian of Eq. (5), we can readily find the dark state is

7 $$|D\rangle =\frac{{{\rm{\Omega }}}_{b}}{{\rm{\Omega }}}|gg\rangle -\frac{{{\rm{\Omega }}}_{a}}{{\rm{\Omega }}}|ee\rangle ,$$

where ${\rm{\Omega }}=\sqrt{{{\rm{\Omega }}}_{a}^{2}+{{\rm{\Omega }}}_{b}^{2}}$. Therefore, we can manipulate the evolution of quantum states with various adiabatic passages.

## Generation of Entangled States

### General adiabatic passage

**Figure 4:**
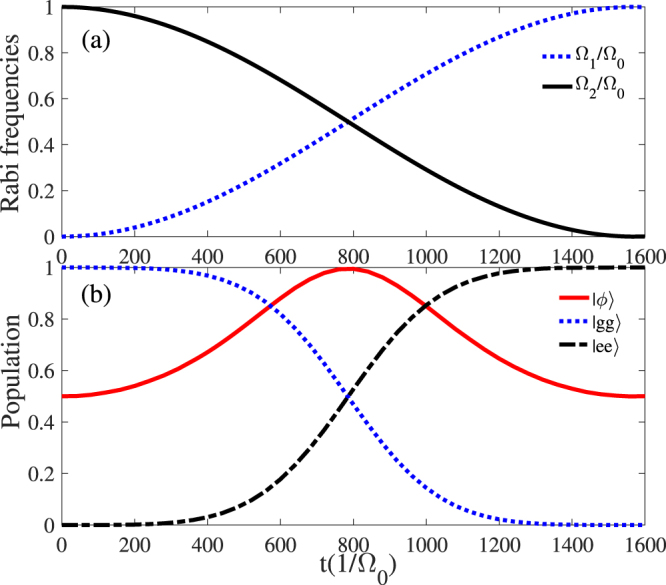
(**a**) Temporal profile of the Rabi frequencies ${{\rm{\Omega }}}_{1}(t){/{\rm{\Omega }}}_{0}$ and ${{\rm{\Omega }}}_{2}(t){/{\rm{\Omega }}}_{0}$. (**b**) The populations of the states $|gg\rangle$, $|ee\rangle$ and $|\varphi \rangle$ versus the interaction time *t*. Other parameters: ${{\rm{\Omega }}}_{1}^{^{\prime} }{/{\rm{\Omega }}}_{0}={{\rm{\Omega }}}_{2}^{^{\prime} }{/{\rm{\Omega }}}_{0}=1$, ${{\rm{\Delta }}}_{1}{/{\rm{\Omega }}}_{0}=20$, ${{\rm{\Delta }}}_{2}{/{\rm{\Omega }}}_{0}=80$, and $\delta {/{\rm{\Omega }}}_{0}=0.1$.

We first utilize the form of sin^2^ (cos^2^) functions^34,35^ to prepare the maximally entangled state $|\varphi \rangle =(|gg\rangle -|ee\rangle )/\sqrt{2}$. The Rabi frequencies ${{\rm{\Omega }}}_{1}(t)$ and ${{\rm{\Omega }}}_{2}(t)$ in the original Hamiltonian ${\hat{H}}_{I}$ are modulated as

8 $${{\rm{\Omega }}}_{1}(t)={{\rm{\Omega }}}_{0}\,\sin \,{(\frac{\delta t}{100})}^{2},\quad {{\rm{\Omega }}}_{2}(t)={{\rm{\Omega }}}_{0}\,\cos \,{(\frac{\delta t}{100})}^{2},$$

where ${{\rm{\Omega }}}_{0}$ is the pulse amplitude, *t* is the operation time. In Fig. 4(a), we plot the Rabi frequencies ${{\rm{\Omega }}}_{1}{/{\rm{\Omega }}}_{0}$ (${{\rm{\Omega }}}_{2}{/{\rm{\Omega }}}_{0}$) versus the interaction time *t* within a quarter period. Figure 4(b) illustrates the populations of the states $|\varphi \rangle$, $|ee\rangle$ and $|gg\rangle$ as ${{\rm{\Omega }}}_{1}^{^{\prime} }{/{\rm{\Omega }}}_{0}={{\rm{\Omega }}}_{2}^{^{\prime} }{/{\rm{\Omega }}}_{0}=1$, ${{\rm{\Delta }}}_{1}{/{\rm{\Omega }}}_{0}=20$, ${{\rm{\Delta }}}_{2}{/{\rm{\Omega }}}_{0}=80$, and $\delta {/{\rm{\Omega }}}_{0}=0.1$. It is easy to find that we can obtain a high population for the state $|\phi \rangle$ at the time *t* = *T*/8 (*T* is pulse period).

### Stimulated Raman adiabatic passage

We choose parameters for the laser pulses suitably to fulfill the boundary condition of the STIRAP

9 $$\mathop{lim}\limits_{t\to -\infty }\frac{{{\rm{\Omega }}}_{a}(t)}{{{\rm{\Omega }}}_{b}(t)}=\mathrm{0,}\,\mathop{lim}\limits_{t\to +\infty }\frac{{{\rm{\Omega }}}_{a}(t)}{{{\rm{\Omega }}}_{b}(t)}=1.$$

**Figure 5:**
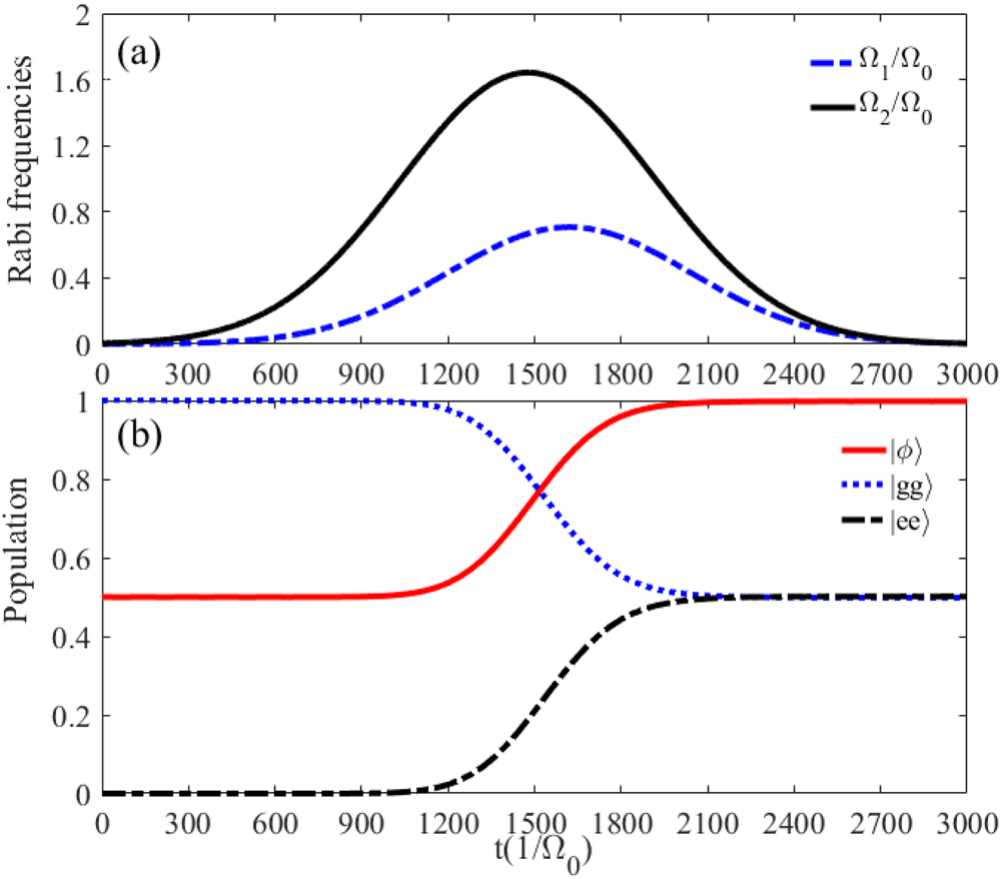
(**a**) Temporal profile of the Rabi frequencies ${{\rm{\Omega }}}_{1}(t){/{\rm{\Omega }}}_{0}$ and ${{\rm{\Omega }}}_{2}(t){/{\rm{\Omega }}}_{0}$. (**b**) The populations of the states $|gg\rangle$, $|ee\rangle$ and $|\varphi \rangle$ versus the interaction time *t*. Other parameters: ${{\rm{\Omega }}}_{1}^{^{\prime} }{/{\rm{\Omega }}}_{0}={{\rm{\Omega }}}_{2}^{^{\prime} }{/{\rm{\Omega }}}_{0}=1$, ${{\rm{\Delta }}}_{1}{/{\rm{\Omega }}}_{0}=20$, ${{\rm{\Delta }}}_{2}{/{\rm{\Omega }}}_{0}=70$, $\delta {/{\rm{\Omega }}}_{0}=1$, $\alpha =\pi /4$, ${t}_{c}=3000{/{\rm{\Omega }}}_{0}$, $T=0.2{t}_{c}$, and $\tau =0.04{t}_{c}$.

Thus, the Rabi frequencies ${{\rm{\Omega }}}_{1}(t)$ and ${{\rm{\Omega }}}_{2}(t)$ in the original Hamiltonian ${\hat{H}}_{I}$ are chosen as

10 $$\begin{array}{rcl}{{\rm{\Omega }}}_{1}(t) & = & {{\rm{\Omega }}}_{0}\,\sin (\alpha )\exp [-\frac{{(t-{t}_{c}/2-\tau )}^{2}}{{T}^{2}}],\\ {{\rm{\Omega }}}_{2}(t) & = & {{\rm{\Omega }}}_{0}\,\cos (\alpha )\exp [-\frac{{(t-{t}_{c}/2-\tau )}^{2}}{{T}^{2}}]\\  &  & +{{\rm{\Omega }}}_{0}\exp [-\frac{{(t-{t}_{c}/2+\tau )}^{2}}{{T}^{2}}],\end{array}$$

where ${{\rm{\Omega }}}_{0}$ is the peak Rabi frequency, ${t}_{c}$ is the pulse duration, and $\tau$ is the delay between the pulses. The shapes of pulses are shown in Fig. 5(a), where the parameters have been chosen as ${t}_{c}=3000{/{\rm{\Omega }}}_{0}$, $T=0.2{t}_{c}$, and $\tau =0.04{t}_{c}$. Figure 5(b) characterizes the populations of states $|\phi \rangle$, $|ee\rangle$ and $|gg\rangle$ corresponding $U{/{\rm{\Omega }}}_{0}=51$, ${{\rm{\Omega }}}_{1}^{^{\prime} }{/{\rm{\Omega }}}_{0}={{\rm{\Omega }}}_{2}^{^{\prime} }{/{\rm{\Omega }}}_{0}=1$, $\delta {/{\rm{\Omega }}}_{0}=1$, $\alpha =\pi /4$, ${t}_{c}=3000{/{\rm{\Omega }}}_{0}$, and $T=0.2{t}_{c}$. It turns out that a longer interaction time is required, i.e. $t=2100{/{\rm{\Omega }}}_{0}$ for achieving the target state, and the population of the target state $|\varphi \rangle$ remains unit when $t\ge 2100{/{\rm{\Omega }}}_{0}$. Compared with the former method, the STIRAP is not restricted to an accurate interaction time but requires a relatively long time.

### Shortcuts to adiabatic passage

In order to obtain the state $|\varphi \rangle$ with STAP, we first consider the case of resonant situation in Eq. (5), i.e. $\nu =\delta +\delta ^{\prime} =0$, then we have

11 $${\hat{H}}_{{\rm{ap}}}(t)={{\rm{\Omega }}}_{a}^{^{\prime} }(t)|rr\rangle \langle gg|+{{\rm{\Omega }}}_{b}^{^{\prime} }(t)|rr\rangle \langle ee|+{\rm{H}}\mathrm{.}{\rm{c}}\mathrm{..}$$

For this effective Hamiltonian, its eigenstates are easily obtained

12 $$|{n}_{0}(t)\rangle =(\begin{array}{c}-\cos \,\theta (t)\\ \sin \,\theta (t)\\ 0\end{array}),\quad |{n}_{\pm }(t)\rangle =\frac{1}{\sqrt{2}}(\begin{array}{c}\sin \,\theta (t)\\ \cos \,\theta (t)\\ \pm 1\end{array}),$$

corresponding eigenvalues ${\varepsilon }_{0}=0$, ${\varepsilon }_{\pm }=\pm \sqrt{2}{\rm{\Omega }}^{\prime} (t)$, respectively, where $\theta (t)=\arctan [{{\rm{\Omega }}}_{a}^{^{\prime} }(t){/{\rm{\Omega }}}_{b}^{^{\prime} }(t)]$, and ${\rm{\Omega }}^{\prime} (t)=\sqrt{{{\rm{\Omega }}}_{a}^{^{\prime} 2}(t)+{{\rm{\Omega }}}_{b}^{^{\prime} 2}(t)}$. The instantaneous eigenstates $|{n}_{k}\rangle$ ($k=\mathrm{0,}\pm \,$) for the effective Hamiltonian ${\hat{H}}_{{\rm{ap}}}(t)$ does not satisfy the Schrödinger equation $i{\partial }_{t}|{n}_{k}\rangle ={\hat{H}}_{{\rm{ap}}}(t)|{n}_{k}\rangle$. According to Berrys transitionless tracking algorithm^36^, we can reverse engineer ${\hat{H}}_{{\rm{cap}}}(t)$ which is related to the original Hamiltonian ${\hat{H}}_{{\rm{ap}}}(t)$, and drive the eigenstates exactly. From refs^37–39^, the simplest Hamiltonian ${\hat{H}}_{{\rm{cap}}}(t)$ is derived in the form

13 $${\hat{H}}_{{\rm{cap}}}(t)=i\sum _{k=\mathrm{0,}\pm }|{\partial }_{t}{n}_{k}(t)\rangle \langle {n}_{k}(t)|=i\dot{\theta }(t)|ee\rangle \langle gg|+{\rm{H}}\mathrm{.}{\rm{c}}\mathrm{.,}$$

where $\dot{\theta }(t)=[{\dot{{\rm{\Omega }}}}_{a}^{^{\prime} }(t){{\rm{\Omega }}}_{b}^{^{\prime} }(t)-{{\rm{\Omega }}}_{a}^{^{\prime} }(t){\dot{{\rm{\Omega }}}}_{b}^{^{\prime} }(t)]/{\rm{\Omega }}^{\prime} {(t)}^{2}$. If the detuning $(\nu =\delta +\delta ^{\prime} \ne \mathrm{0)}$ is considered as shown in Eq. (5), we can adiabatically eliminate the terms of $|rr\rangle$ state under the large detuning condition $\nu \gg \{{{\rm{\Omega }}}_{a},{{\rm{\Omega }}}_{b}\}$, leading to the effective Hamiltonian

14 $${\hat{H}}_{{\rm{e}}{\rm{f}}{\rm{f}}}=\frac{{{\rm{\Omega }}}_{a}^{2}}{\nu }|gg\rangle \langle gg|+\frac{{{\rm{\Omega }}}_{b}^{2}}{\nu }|ee\rangle \langle ee|+\frac{{{\rm{\Omega }}}_{a}{{\rm{\Omega }}}_{b}}{\nu }|gg\rangle \langle ee|+{\rm{H}}\mathrm{.}{\rm{c}}\mathrm{..}$$

Then we choose ${{\rm{\Omega }}}_{a}=i{{\rm{\Omega }}}_{b}=\tilde{{\rm{\Omega }}}$ in order to cancel the first two terms, and the final Hamiltonian becomes

15 $${\tilde{H}}_{{\rm{eff}}}=i\frac{{\tilde{{\rm{\Omega }}}}^{2}}{\nu }|gg\rangle \langle ee|+{\rm{H}}\mathrm{.}{\rm{c}}\mathrm{.,}$$

where $$\tilde{{\rm{\Omega }}}=\sqrt{\nu \dot{\theta }(t)}=\sqrt{\nu [{\dot{{\rm{\Omega }}}}_{a}^{^{\prime} }(t){{\rm{\Omega }}}_{b}^{^{\prime} }(t)-{\dot{{\rm{\Omega }}}}_{b}^{^{\prime} }(t){{\rm{\Omega }}}_{a}\text{'}(t)]/{\rm{\Omega }}^{\prime} {(t)}^{2}}$$.

**Figure 6:**
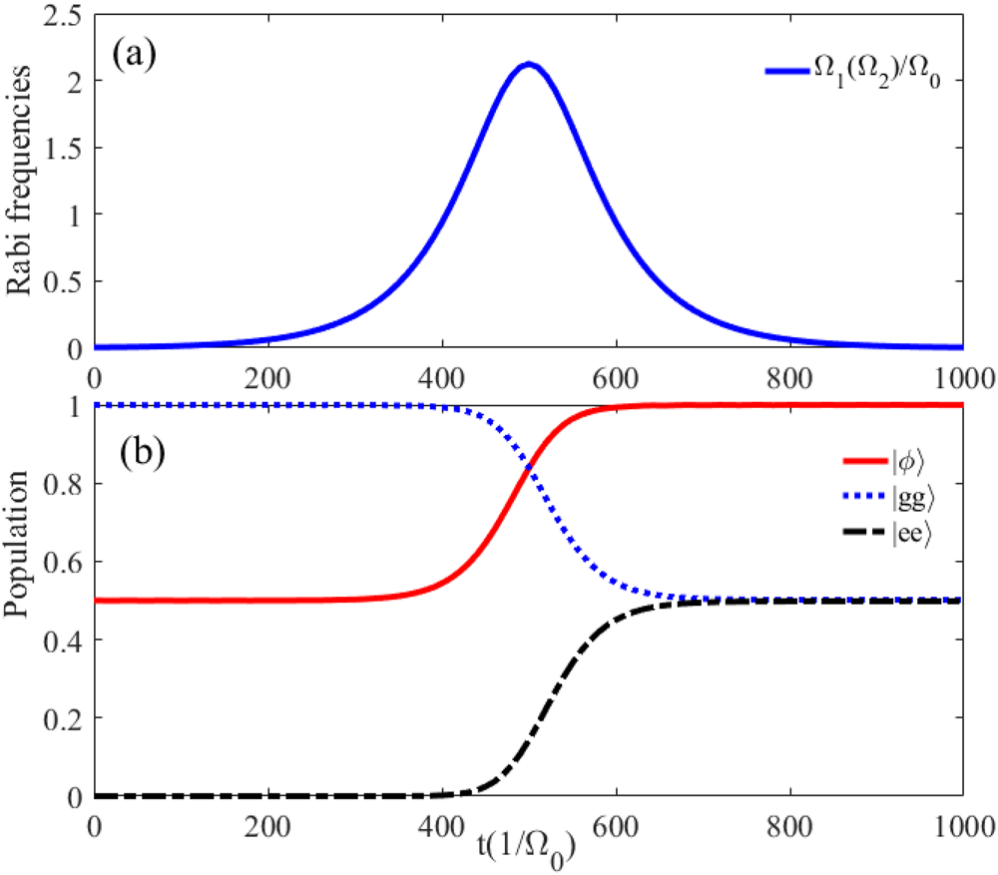
(**a**) Temporal profile of the Rabi frequencies ${{\rm{\Omega }}}_{1}(t){/{\rm{\Omega }}}_{0}$ and ${{\rm{\Omega }}}_{2}(t){/{\rm{\Omega }}}_{0}$. (**b**) The populations of the states $|gg\rangle$, $|ee\rangle$ and $|\varphi \rangle$ versus the interaction time *t*. Other parameters: ${{\rm{\Omega }}}_{1}^{^{\prime} }{/{\rm{\Omega }}}_{0}={{\rm{\Omega }}}_{2}^{^{\prime} }{/{\rm{\Omega }}}_{0}=1$, ${{\rm{\Delta }}}_{1}{/{\rm{\Omega }}}_{0}=20$, ${{\rm{\Delta }}}_{2}{/{\rm{\Omega }}}_{0}=68$, ${t}_{c}=1000{/{\rm{\Omega }}}_{0}$, $T=0.12{t}_{c}$, and $\tau =0.1{t}_{c}$.

We will show below the numerical analysis of the creating the two-atom Bell state governed by the STAP. Here the Rabi frequencies ${{\rm{\Omega }}}_{a}^{^{\prime} }(t)$ and ${{\rm{\Omega }}}_{b}(t)^{\prime}$ in the Hamiltonian ${\hat{H}}_{{\rm{ap}}}$ are chosen as

16 $${{\rm{\Omega }}}_{a}^{^{\prime} }(t)={{\rm{\Omega }}}_{0}\exp [-\frac{(t-{t}_{c}/2-\tau )}{{T}^{2}}]$$

and

17 $${{\rm{\Omega }}}_{b}^{^{\prime} }(t)={{\rm{\Omega }}}_{0}\exp [-\frac{(t-{t}_{c}/2+\tau )}{{T}^{2}}],$$

where ${{\rm{\Omega }}}_{0}$ is the pulse amplitude. The forms of above pulses just correspond to ${{\rm{\Omega }}}_{1}(t)=i{{\rm{\Omega }}}_{2}(t)=-28\tilde{{\rm{\Omega }}}$ for the original Hamiltonian ${\hat{H}}_{I}$ of Eq. (1). In Fig. 6(a), we plot the pulses with the operation time ${t}_{c}=1000{/{\rm{\Omega }}}_{0}$, $T=0.12{t}_{c}$ and $\tau =0.1{t}_{c}$. Figure 6(b) shows the populations of state $|\phi \rangle$, $|ee\rangle$ and $|gg\rangle$ corresponding $U{/{\rm{\Omega }}}_{0}=51$, ${{\rm{\Omega }}}_{1}^{^{\prime} }{/{\rm{\Omega }}}_{0}={{\rm{\Omega }}}_{2}^{^{\prime} }{/{\rm{\Omega }}}_{0}=1$, $\delta {/{\rm{\Omega }}}_{0}=1$, ${t}_{c}=1000{/{\rm{\Omega }}}_{0}$, and $T=0.12{t}_{c}$, $\tau =0.1{t}_{c}$. Compared with the former two methods, this STAP-based entanglement generation requires neither a long time nor an acurate interaction time.

## Discussion

**Figure 7:**
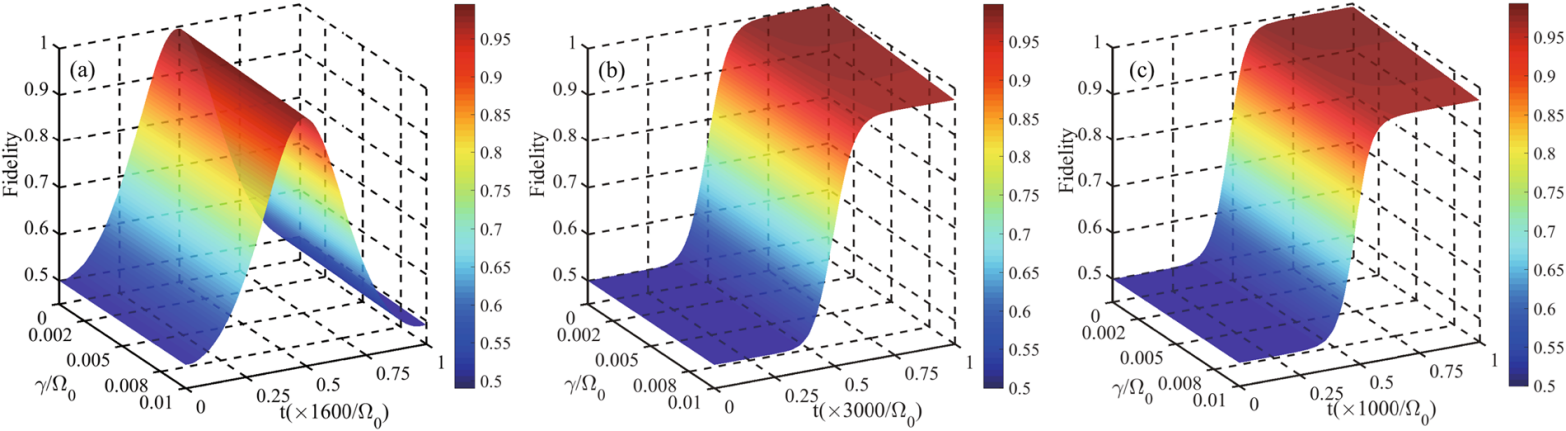
The fidelity for the state $|\varphi \rangle$ as a function of $\gamma {/{\rm{\Omega }}}_{0}$ and interaction time *t* with ${{\rm{\Omega }}}_{1}^{^{\prime} }{/{\rm{\Omega }}}_{0}=1$, ${{\rm{\Omega }}}_{2}^{^{\prime} }{/{\rm{\Omega }}}_{0}=1$, ${{\rm{\Delta }}}_{1}{/{\rm{\Omega }}}_{0}=20$, $U={{\rm{\Delta }}}_{2}-{{\rm{\Delta }}}_{1}+\delta$, governed by the original Hamiltonian ${\hat{H}}_{I}$. (**a**) The parameters are chosen as $\delta {/{\rm{\Omega }}}_{0}=0.1$ and ${{\rm{\Delta }}}_{2}{/{\rm{\Omega }}}_{0}=80$. (**b**) The parameters are chosen as $\delta {/{\rm{\Omega }}}_{0}=1$, ${{\rm{\Delta }}}_{2}{/{\rm{\Omega }}}_{0}=70$, $\alpha =\pi /4$, ${t}_{c}=3000{/{\rm{\Omega }}}_{c}$, $T=0.2{t}_{c}$, and $\tau =0.04{t}_{c}$. (**c**) The parameters are chosen as $\delta {/{\rm{\Omega }}}_{0}=1$, ${{\rm{\Delta }}}_{2}{/{\rm{\Omega }}}_{0}=68$, ${t}_{c}=3000{/{\rm{\Omega }}}_{c}$, $T=0.2{t}_{c}$, and $\tau =0.04{t}_{c}$.

We have illustrated how to prepare the maximally entangled state $(|gg\rangle -|ee\rangle )/\sqrt{2}$ in the ideal situation by manipulating pulses in different ways. However, the actual system will interact with the environment inevitably, which affects the availability of these methods. Thus it is necessary to investigate the influence of spontaneous emission of atoms on our proposal. When the dissipation is considered, the evolution of the system can be modeled by a master equation in Lindblad form^40,41^

18 $$\dot{\hat{\rho }}=-i[{\hat{H}}_{I},\hat{\rho }]-\frac{1}{2}\sum _{n=1}^{4}[{\hat{ {\mathcal L} }}_{n}^{\dagger }{\hat{ {\mathcal L} }}_{n}\hat{\rho }-2{\hat{ {\mathcal L} }}_{n}\hat{\rho }{\hat{ {\mathcal L} }}_{n}^{\dagger }+\hat{\rho }{\hat{ {\mathcal L} }}_{n}^{\dagger }{\hat{ {\mathcal L} }}_{n}],$$

where $\rho$ is the density matrix of the whole system and ${\hat{ {\mathcal L} }}_{1}=\sqrt{\gamma /2}|g{\rangle }_{1}\langle r|$, ${\hat{ {\mathcal L} }}_{2}=\sqrt{\gamma /2}|e{\rangle }_{1}\langle r|$, ${\hat{ {\mathcal L} }}_{3}=\sqrt{\gamma /2}|g{\rangle }_{2}\langle r|$, and ${\hat{ {\mathcal L} }}_{4}=\sqrt{\gamma /2}|e{\rangle }_{2}\langle r|$ are Lindblad operators describing the dissipative processes, and *γ* denotes the atomic decay rate. For the sake of convenience, we have assumed the Rydberg state $|r\rangle$ can decay towards the two ground states $|g\rangle$ and $|e\rangle$ with equal spontaneous emission rate. The state $|\varphi \rangle$ can act as the ideally final state to check the performance of our scheme, thus we adopt the definition of population to assess the fidelity $F=P=\langle \varphi |\hat{\rho }(t)|\varphi \rangle$. In Fig. 7, we plot the fidelity of the target state as a function of $\gamma {/{\rm{\Omega }}}_{0}$ and the interaction time *t* with ${{\rm{\Omega }}}_{1}^{^{\prime} }{/{\rm{\Omega }}}_{0}=1$, ${{\rm{\Omega }}}_{2}^{^{\prime} }{/{\rm{\Omega }}}_{0}=1$, ${{\rm{\Delta }}}_{1}{/{\rm{\Omega }}}_{0}=20$, $U={{\rm{\Delta }}}_{2}-{{\rm{\Delta }}}_{1}+\delta$.

In Fig. 7(a), we can see that the fidelity is immune to the spontaneous emission of atoms, and when we choose $\delta {/{\rm{\Omega }}}_{0}=0.1$, ${{\rm{\Delta }}}_{2}{/{\rm{\Omega }}}_{0}=80$ and $\gamma {/{\rm{\Omega }}}_{0}=0.01$, the fidelity remains 98.5%. Since the population of the state $|rr\rangle$ is near to zero all the time, the spontaneous emission has little influence on the fidelity. Figure 7(b) shows a high fidelity 97.5% with $\delta /{{\rm{\Omega }}}_{0}=1$, $\alpha =\pi /4$, ${{\rm{\Delta }}}_{2}/{{\rm{\Omega }}}_{0}=70$ and $\gamma /{{\rm{\Omega }}}_{0}=0.01$. In addition, in Fig. 7(c), a high fidelity 97.3% can be obtained when the parameters are chosen as $\delta /{{\rm{\Omega }}}_{0}=1$, ${{\rm{\Delta }}}_{2}/{{\rm{\Omega }}}_{0}=68$, and $\gamma /{{\rm{\Omega }}}_{0}=0.01$.

In experiments, the ground-state antiblockade model can be realized in ^87^Rb atoms which are trapped in two tightly focused dipole traps^21,42^. The ground state $|g\rangle$ corresponds to $\mathrm{|5}{S}_{1/2},\,F=\mathrm{1,}\,{M}_{F}=2\rangle$ and the ground state $|e\rangle$ corresponds to $\mathrm{|5}{S}_{1/2},\,F=\mathrm{2,}\,{M}_{F}=2\rangle$, the Rydberg state corresponds to $|r\rangle \equiv \mathrm{|58}{D}_{\mathrm{3/2}},\,F=\mathrm{3,}\,{M}_{F}=3\rangle$, respectively. The atoms are excited to the Rydberg state by a two-photon transition, and the resulting order of magnitude of Rabi frequency ${{\rm{\Omega }}}_{2^{\prime} }$ for atom 1 and the Rabi frequency ${{\rm{\Omega }}}_{1^{\prime} }$ for atom 2 is about ${{\rm{\Omega }}}_{0}=2\pi \times 6.8$ MHz. The spontaneous emission rate from the Rydberg state is $\gamma =2\pi \times 4.8$ kHz. By substituting these values into the master equation, we find the fidelities of generating two-atom entanglement with the above three methods are all beyond 99%.

In summary, we have put forward an efficient scheme for the ground-state antiblockade of Rydberg atoms and prepare two-atom entangled state. Three kinds of pulses are exploited to obtain the maximally entangled state, and a high fidelity is achievable with the current experimental parameters. Most interestingly, this process is robust against the decoherence induced by spontaneous emission of atoms. We hope that our scheme could find some applications in the near future.

## Methods

### Calculation of effective coupling strength

From Eqs (1) to (2), the effective coupling strength calculated by the 2nd-order perturbation theory are:

19 $$\begin{array}{c}\{\begin{array}{c}\frac{\langle gg|H|gr\rangle \langle gr|H|gg\rangle }{{{\rm{\Delta }}}_{2}}=\frac{{{\rm{\Omega }}}_{1}^{^{\prime} 2}}{{{\rm{\Delta }}}_{2}}\\ \frac{\langle rr|H|gr\rangle \langle gr|H|gg\rangle }{{{\rm{\Delta }}}_{2}}=\frac{{{\rm{\Omega }}}_{1}{{\rm{\Omega }}}_{11}}{{{\rm{\Delta }}}_{1}}{e}^{i\delta t},\\ \frac{\langle rr|H|gr\rangle \langle gr|H|rr\rangle }{{{\rm{\Delta }}}_{2}}=\frac{{{\rm{\Omega }}}_{1}^{2}}{{{\rm{\Delta }}}_{2}}\end{array}\quad \,\{\begin{array}{c}\frac{\langle gg|H|rg\rangle \langle rg|H|gg\rangle }{-{{\rm{\Delta }}}_{1}}=\frac{{{\rm{\Omega }}}_{1}^{2}}{-{{\rm{\Delta }}}_{1}}\\ \frac{\langle rr|H|rg\rangle \langle rg|H|gg\rangle }{-{{\rm{\Delta }}}_{1}}=\frac{{{\rm{\Omega }}}_{1}{{\rm{\Omega }}}_{11}}{-{{\rm{\Delta }}}_{1}}{e}^{i\delta t},\\ \frac{\langle rr|H|gr\rangle \langle rg|H|rr\rangle }{{{\rm{\Delta }}}_{1}}=\frac{{{\rm{\Omega }}}_{2}^{^{\prime} 2}}{-{{\rm{\Delta }}}_{1}}\end{array}\\ \{\begin{array}{c}\frac{\langle ee|H|er\rangle \langle er|H|ee\rangle }{-{{\rm{\Delta }}}_{1}}=\frac{{{\rm{\Omega }}}_{2}^{2}}{-{{\rm{\Delta }}}_{1}}\\ \frac{\langle rr|H|er\rangle \langle er|H|ee\rangle }{-{{\rm{\Delta }}}_{1}}=\frac{{{\rm{\Omega }}}_{2}{{\rm{\Omega }}}_{2}^{^{\prime} }}{{{\rm{\Delta }}}_{1}}{e}^{i\delta t},\\ \frac{\langle rr|H|er\rangle \langle er|H|rr\rangle }{{{\rm{\Delta }}}_{1}}=\frac{{{\rm{\Omega }}}_{2}^{2}}{{{\rm{\Delta }}}_{1}}\end{array}\quad \,\,\,\{\begin{array}{c}\frac{\langle ee|H|re\rangle \langle re|H|ee\rangle }{-{{\rm{\Delta }}}_{2}}=\frac{{{\rm{\Omega }}}_{2}^{^{\prime} 2}}{{{\rm{\Delta }}}_{2}}\\ \frac{\langle rr|H|re\rangle \langle re|H|ee\rangle }{{{\rm{\Delta }}}_{2}}=\frac{{{\rm{\Omega }}}_{2}{{\rm{\Omega }}}_{2}^{^{\prime} }}{{{\rm{\Delta }}}_{2}}{e}^{i\delta t},\\ \frac{\langle rr|H|re\rangle \langle re|H|rr\rangle }{{{\rm{\Delta }}}_{2}}=\frac{{{\rm{\Omega }}}_{2}^{2}}{{{\rm{\Delta }}}_{2}}\end{array}\end{array}$$

### Cancellation of ground-state Stark shifts

**Figure 8:**
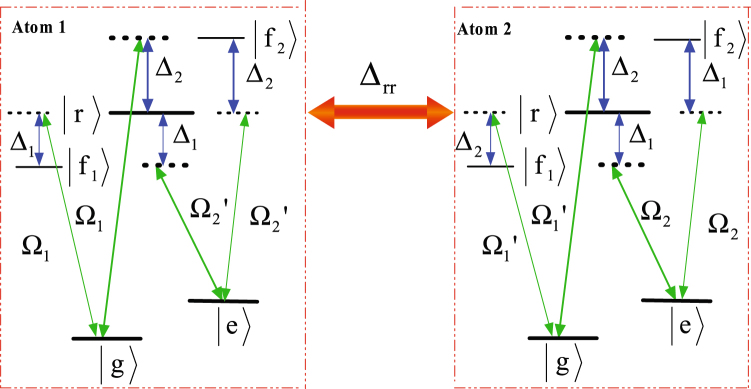
Schematic view of eliminating the Stark shifts of the states $|gg\rangle$ and $|ee\rangle$ by introducing two auxiliary levels $|{f}_{1}\rangle$ and $|{f}_{2}\rangle$.

From Eqs (3) to (4), the Stark shifts of states $|gg\rangle$ and $|ee\rangle$ can be eliminated by introducing two auxiliary levels $|{f}_{1}\rangle$ and $|{f}_{2}\rangle$, as shown in Fig. 8. The transition $|gg\rangle (|ee\rangle )\leftrightarrow |rr\rangle$ is driven by a another classical laser field with the Rabi frequency ${{\rm{\Omega }}}_{a}({{\rm{\Omega }}}_{b})$ with the detuning $-(\delta +\delta ^{\prime} )$, thus, leading to the Stark shifts of states $|gg\rangle$ and $|ee\rangle$ are $-({{\rm{\Omega }}}_{1}^{^{\prime} 2}{/{\rm{\Delta }}}_{2}-{{\rm{\Omega }}}_{1}^{2}{/{\rm{\Delta }}}_{1})$ and $-({{\rm{\Omega }}}_{2}^{^{\prime} 2}{/{\rm{\Delta }}}_{2}-{{\rm{\Omega }}}_{2}^{2}{/{\rm{\Delta }}}_{1})$, respectively. Therefore, the whole Stark shifts of states $|gg\rangle$ and $|ee\rangle$ can be eliminated.

### Generation of antisymmetric Bell state

**Figure 9:**
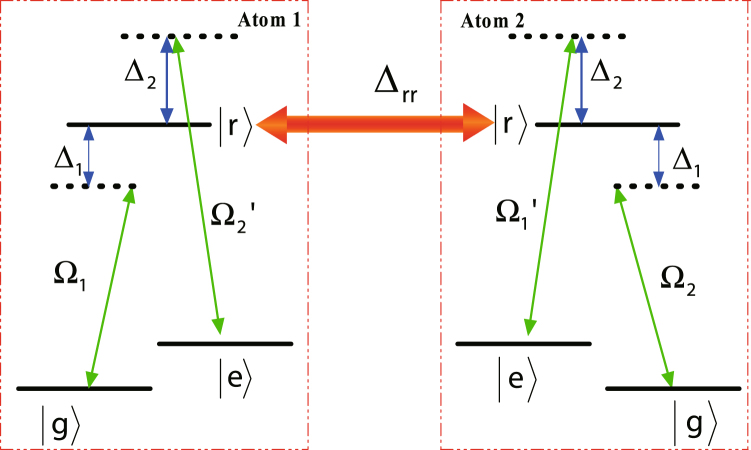
Schematic view of atomic-level configuration for the generation of antisymmetric Bell state. $|r\rangle$ is the Rydberg state, while $|g\rangle$ and $|e\rangle$ are two ground states. ${{\rm{\Delta }}}_{rr}$ denotes the RRI strength. For atom 1, the transition $|g\rangle \leftrightarrow |r\rangle$ is driven by a classical laser field with Rabi frequency ${{\rm{\Omega }}}_{1}$ and the transition $|e\rangle \leftrightarrow |r\rangle$ is driven by a classical laser field with Rabi frequency ${{\rm{\Omega }}}_{2}^{^{\prime} }$. For atom 2, the transition $|g\rangle \leftrightarrow |r\rangle$ is driven by a classical laser field with Rabi frequency ${{\rm{\Omega }}}_{2}$ and the transition $|e\rangle \leftrightarrow |r\rangle$ is driven by a classical laser field with Rabi frequency ${{\rm{\Omega }}}_{1}^{^{\prime} }$. ${{\rm{\Delta }}}_{\mathrm{1(2)}}$ represents the corresponding detuning parameter.

When we consider two identical atoms as shown in Fig. 9, the effective Hamiltonian can be obtained as

20 $$\begin{array}{rcl}{\hat{H}}_{AE} & = & (\frac{{{\rm{\Omega }}}_{1}^{2}}{{{\rm{\Delta }}}_{1}}+\frac{{{\rm{\Omega }}}_{2}^{2}}{{{\rm{\Delta }}}_{1}}-\frac{{{\rm{\Omega }}}_{2}^{^{\prime} 2}}{{{\rm{\Delta }}}_{2}}-\frac{{{\rm{\Omega }}}_{1}^{^{\prime} 2}}{{{\rm{\Delta }}}_{2}})|rr\rangle \langle rr|\\  &  & +[(\frac{{{\rm{\Omega }}}_{2}{{\rm{\Omega }}}_{2}^{^{\prime} }}{{{\rm{\Delta }}}_{2}}-\frac{{{\rm{\Omega }}}_{2}{{\rm{\Omega }}}_{2}^{^{\prime} }}{{{\rm{\Delta }}}_{1}}){e}^{i\delta t}|rr\rangle \langle eg|\\  &  & +(\frac{{{\rm{\Omega }}}_{1}{{\rm{\Omega }}}_{2}^{^{\prime} }}{{{\rm{\Delta }}}_{2}}-\frac{{{\rm{\Omega }}}_{1}{{\rm{\Omega }}}_{1}^{^{\prime} }}{{{\rm{\Delta }}}_{1}}){e}^{i\delta t}|rr\rangle \langle ge|+{\rm{H}}\mathrm{.}{\rm{c}}\mathrm{.}]\mathrm{.}\end{array}$$

Thus, we can also use this effective Hamiltonian to prepare the entangled state $(|ge\rangle -|eg\rangle )/\sqrt{2}$.
